# “An apple pie a day does not keep the doctor away”: Fictional depictions of gout in contemporary film and television

**DOI:** 10.1186/s41927-020-00174-z

**Published:** 2021-01-18

**Authors:** Christina Derksen, Rachel Murdoch, Keith J. Petrie, Nicola Dalbeth

**Affiliations:** 1 Health Psychology and Behavioral Medicine, Jacobs University Bremen, Bremen, Germany; 2 Department of Medicine, Faculty of Medical and Health Sciences, University of Auckland, 85 Park Rd, Grafton, Auckland, New Zealand; 3 Department of Psychological Medicine, University of Auckland, Auckland, New Zealand

**Keywords:** Gout, Arthritis, Communications media

## Abstract

**Background:**

Fictional portrayals of illness and medical management in film and television can reflect and perpetuate cultural stereotypes about illness. The aim of this study was to analyse fictional depictions of gout in contemporary film and television.

**Methods:**

We conducted a search for English language depictions of gout in film and television since 1990 using the Internet Movie Database (IMDb), other internet media databases, and member suggestions from the Gout, Hyperuricemia and Crystal-Associated Disease Network (G-CAN). Film and television episodes with gout content were analysed for depictions of characters with gout, causal factors, and management strategies (*n*=44).

**Results:**

Gout was used to denote royalty or nobility in historical settings, and as a plot device to explain the absence of characters from key events. The most commonly depicted causes of gout were overindulgence of food and alcohol (61%), and portrayals of biological causes were infrequent (12%). Common management strategies were change in diet (36%) and short-term pain relief (32%), with only one mention of urate-lowering therapy (5%). The majority of films and television episodes depicted gout as humorous (59%) and embarrassing (50%).

**Conclusions:**

In contemporary film and television, gout is portrayed as a humorous and embarrassing condition, caused by dietary indulgence. These depictions may reinforce inaccurate beliefs about the causes of gout and its management.

**Supplementary Information:**

The online version contains supplementary material available at 10.1186/s41927-020-00174-z.

## Background

Gout is a chronic disease of monosodium urate (MSU) crystal deposition that presents as recurrent flares of painful and disabling arthritis [1]. The disease has a strong genetic basis and more frequently affects older people, men, and those with kidney disease, diabetes, and heart disease [2]. The central strategy for effective gout management is long-term urate-lowering therapy in order to reduce serum urate; long-term, this strategy leads to MSU crystal dissolution, suppression of gout flares and regression of tophi [3]. Gout prevalence is increasing, but low initiation and persistence of urate-lowering therapy and low achievement of serum urate targets remain challenging [4].

Consistent with depictions of the illness in historical art and literature [5], gout is widely viewed in contemporary society as a humorous, self-inflicted condition caused by dietary excess [6]. These views can contribute to stigma and embarrassment about gout [7], and influence patients’ lay beliefs or illness perceptions [8]. Inaccurate beliefs about gout can also lead to ineffective management strategies, and contribute to worse outcomes [8, 9].

Film and television reach a broad range of society and therefore have the potential to contribute substantially to illness beliefs. Analysis of fictional medical dramas on television has shown that these programmes depict a wide range of common medical conditions [10]. However, depictions of illness and medical management in film and television may contribute to cultural stereotypes about an illness. For example, analyses of depictions of mental illness have shown that on-screen portrayals are negative and stigmatizing, and may influence people with mental illness seeking appropriate help [11–13]. Depictions of epilepsy [14], medication use [15], infectious diseases [16], dermatology [17] and termination of pregnancy [18] may be inaccurate. The aim of this study was to analyse fictional depictions of gout and its management in contemporary film and television.

## Methods

### Data sources and searches

We conducted a content analysis of gout depictions in film and television. The “Internet Movie Database” (IMDb), which has been used to identify content in other scientific studies [14, 19], was selected as the main database. The keyword “gout” was used as a search term for keywords (inserted for a movie by viewers in the community), titles, plot descriptions, film quotes and trivia. As secondary databases, the “Literature, Arts & Medicine Data Base”, “allmovie.com”, “reel.com”, “rottentomatoes.com”, “ForeignFilms”, the “Chinese database”, “Asian database” and “Bollywood Movie Database” were searched. The keywords “gout film”, “gout movie”, “movie showing/ depicting gout”, “film showing/ depicting gout”, “film character with gout”, “movie character with gout”, “TV show gout” and “TV show character with gout” were used for Google online searches.

Requests to nominate films and television episodes were posted on the IMDb community pages and emailed to experts from the international volunteer organization Gout, Hyperuricemia and Crystal-Associated Disease Network (G-CAN) via the G-CAN mailing list.

### Inclusion criteria

Films and television episodes were included in the analysis if they were fictional, released after 1990, produced in English, and had content related to gout in humans. Documentary and reality films and television episodes were excluded.

### Data extraction and content analysis

Each film or television episode was watched in full by two researchers (CD and RM). Where available, films and episodes were watched with closed captions. All text relevant to gout was extracted and transcribed in script form with relevant time stamps by one of the two researchers and was checked by the other researcher.

The films and television episodes were coded by both researchers at the time of viewing, and relevant text extracts were reviewed. The following data were collected: media type (TV/film), genre (e.g. comedy, drama), setting (historical or modern-day), year of production, country depicted and characteristics of the affected person (age, sex, role, member of the nobility or aristocracy, relationship to other characters, comorbid conditions including being overweight or obese). When the same character with gout appeared in more than one television episode, each episode was coded individually. The character’s experience of gout (for example, as painful, affecting mobility, impacting on ability to attend events and affecting ability to work) was also recorded. Causes of gout were recorded and classified into biological factors (such as genetics and kidney dysfunction), ageing, obesity, and overindulgence of food, alcohol and sugary drinks. Management of gout was recorded and coded into urate-lowering therapy, serum urate monitoring, pain medication, anti-inflammatory medication, and dietary strategies. Reference to gout as a humorous topic and as a source of social embarrassment was recorded. Discordant coding between the two researchers (11% of results, primarily for comorbid conditions) was resolved by discussion and review with a third author (ND).

### Statistical analyses

Analysis was primarily descriptive. Percentages for the numerical coding were calculated. In addition to all content, depictions in historical and modern-day settings are reported separately.

## Results

### Search results

**Fig. 1:**
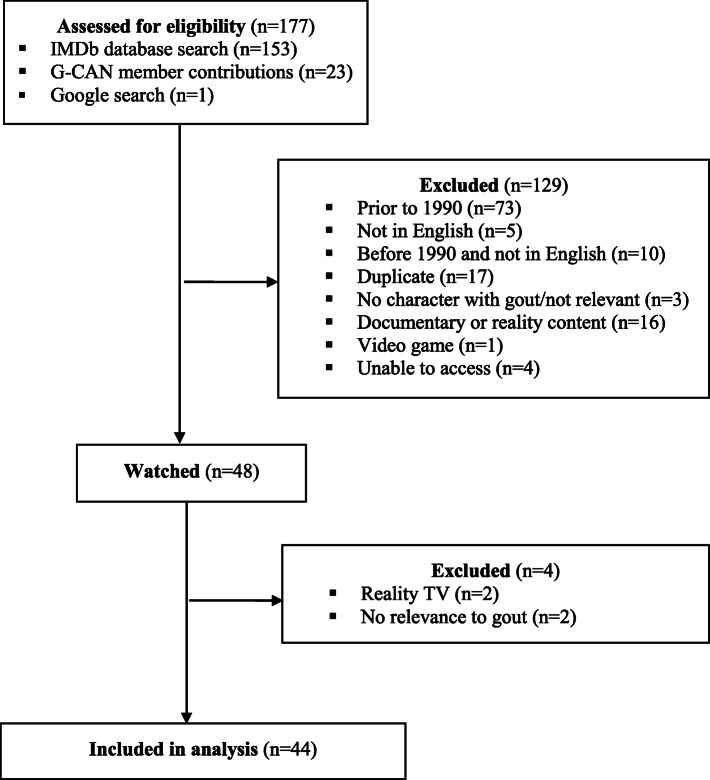
Search Results

Figure 1 shows the search results. The IMDb search resulted in 153 results, with an additional 23 results from G-CAN member input, and one additional result from a Google search. No results were obtained from the other database searches. There were 129 films and television episodes excluded; 83 due to being released prior to 1990, 17 were duplicate results, 16 were documentary or reality content, five were not available in English, and 10 were released before 1990 and not available in English. The majority of content included in the analysis were television episodes (29/44, 66%) with the rest films (15/44, 34%). One television episode had two characters with gout; these characters were analysed separately making the overall number of depictions analysed 45, from 44 films and television episodes. Settings were both historical (20/44, 45%) and modern-day (24/44, 55%), and most were either drama (22/44, 50%) or comedy (17/44, 39%) genres. The full list of films and television episodes included in the study are listed in the supplementary material.

### Character features and experiences of gout

There were 37 depictions of people with gout (Table 1). The majority of characters with gout were male (32/37, 86%), and a protagonist (25/37, 68%). Most were depicted as having comorbidities (22/37, 59%), such as being overweight or obese (14/37, 38%). The character with gout was shown as ageing in 13/37 (35%) of depictions. In historical settings, gout was commonly used as a method of depicting members of the nobility, with 11/18 (61%) of characters with gout being noble or aristocratic. In contrast, in modern-day settings, only 1/19 (5%) characters with gout were noble or aristocratic.

**Table 1:** Fictional depictions of characters with gout in film and television episodes with characters with gout (*n*=36 films and television episodes; one episode had two characters with gout)

	Total ***N***=37	Historical setting ***N***=18^**a**^	Modern-day setting ***N***=19
Male	32/37, 86%	16/18, 89%	16/19, 84%
Female	5/37, 14%	2/18, 11%	3/19, 16%
Protagonist	25/37, 68%	9/18, 50%	16/19, 84%
Antagonist	10/37, 27%	6/18, 33%	4/19, 21%
Neither protagonist nor antagonist	2/37, 5%	2/18, 11%	0, 0%
Character has comorbidities	22/37, 59%	11/18, 61%	11/19, 58%
Overweight/obese	14/37, 38%	6/18, 33%	8/19, 42%
Old age	13/37, 35%	6/18, 33%	7/19, 37%
Noble person/aristocrat	12/37, 32%	11/18, 61%	1/19, 5%
Gout painful	28/37, 76%	15/18, 83%	13/19, 68%
Gout affects mobility	16/37, 43%	12/18, 67%	4/19, 21%
Gout prevents attendance at an event	8/37, 22%	3/18, 17%	5/19, 26%
Gout affects work	5/37, 14%	2/18, 11%	3/19, 16%

^a^One episode had two characters with gout, these were counted separately

Gout was depicted as painful (28/37, 76%), affecting mobility (16/37, 43%), as a reason or excuse to prevent attendance at social functions or activities (8/37, 22%) and as affecting work (5/37, 14%); for example, in Doctors, Charlie was unable to attend an audition due to a gout flare, “Ow! It’s painful … This toe will not be tapping today” (Doctors, S11Ep234, BBC).

### Gout as a narrative device

Gout was commonly used to set the scene of nobility, particularly in historical settings. In Vatel, the Prince has an attack of gout just as the King arrives to visit: “We have to be perfect! Go, go, go, go”, Vatel: “The Prince’s gout is bad. Tell Dr. Bourdelot” (Vatel, 2000, Légende Films). The association of gout with nobility was used for humorous effect in some modern-day depictions, such as in Keeping Up Appearances, where it was used to demonstrate Hyacinth’s desire to be seen as part of the upper classes, as she convinces her husband to pretend to have gout rather than a fungus infection as it is “an affliction acceptable in the very highest circles.” (Keeping Up Appearances, S4Ep9, BBC).

Having gout was also used as a method of emphasising character flaws such as weakness, greed and overindulgence. In Game of Thrones, the character with gout, Prince Doran, is murdered by Ellaria, who says “When was the last time you left this palace? You don’t know your own people, their disgust for you … Your son is weak, just like you. And weak men will never rule Dorne again.” (Game of Thrones, S6Ep1, HBO).

Gout was used as a plot device to explain a character’s absence from important events. An example of this is in The Favourite, where the Queen is portrayed as a self-indulgent and weak ruler, trying to avoid her responsibilities. She does not attend important meetings with officials due to attacks of gout, causing others to use her absence to advance their political ambitions: “Might I remind you you’re not the Queen?” Sarah: “No, she has sent me to speak for her. She’s unwell.” (The Favourite, 2018, Fox Searchlight Pictures). This device is also used in Marco Polo where Kublai Khan can’t attend a feast due to an attack of gout, sending his son in his place, Jingim: “Hobbled by gout … He sends his regrets and me in his stead.” (Marco Polo, S1Ep3, Netflix).

### Depictions of the causes of gout

Causes with examples are shown in Table 2. For those 33 films and television episodes depicting a cause of gout, overindulgence was portrayed as a cause in 20 (61%), for example in Keeping Up Appearances it is described as caused by an “excess of good living” (Keeping Up Appearances, S4Ep9, BBC), and in Marco Polo by “an excess of wine” (Marco Polo, S1Ep3, Netflix). Obesity was depicted in 14/33 (42%), as in Disenchantment: “A disease of kings, you know. Successful kings, that is. A plump, disease-ridden body is a sign you’ve really made it in modern society” (Disenchantment, S2Ep7, Netflix). Biological causes were portrayed in 4/33 (12%) and genetic causes in 3/33 (9%) of films and television episodes portraying a causal factor.

**Table 2:** Causes of gout in films and television episodes in which causes were depicted (*n*=33 films and television episodes)

Cause of gout	Total ***N***=33	Historical setting ***N***=14^**a**^	Modern-day setting ***N***=19	Examples
Overindulgence of food and alcohol	20/33, 61%	8/14, 57%	12/19, 63%	**Alf Roberts:** “Saying it’s all my own fault, eating the wrong food, drinking too much, carrying too much weight.” (Coronation Street, S1Ep3641, ITV)
				**Colleague of Dr Charlie Bradford**: “Couldn’t you have laid off the red wine and overindulgence for one night?” (Doctors, S11Ep234, BBC)
Obesity	14/33, 42%	6/14, 43%	8/19, 42%	**Reginald Rodgers: “**Mainly obese men with fatty rich diets.” (Gut punch, S1Ep1)
				**Mrs Wolowitz**: “How can one little toe hurt so bad?”. **Howard Wolowitz:** “Maybe because that little piggy is being crushed by the barn!” (The Big Bang Theory, S7Ep9, CBS)
Ageing	13/33, 39%	6/14, 43%	7/19, 37%	**Hank Hill**: “Gout … that’s an old man’s disease.” (King of the Hill, S3Ep18, Fox Network) **Dana Scully**: “Presbyopia’s a natural part of the ageing process. We’re all gonna go through it, Mulder. Just wait til you get gout. Gout!” (The X-Files, S11Ep9, Fox Network)
Biological	4/33, 12%	0, 0%	4/19, 21%	**Monica Geller:** “Do you, or any of your blood relatives, have … gout?” (Friends, S9Ep22, NBC)

^a^One episode had two characters with gout, these were counted separately

Specific foods portrayed as associated with gout are shown in Table 3, including different types of meat, alcoholic beverages and food such as “drinkable cheese” (Disenchantment S2Ep7, Netflix).

**Table 3:** Depictions of specific dietary associations with gout (*n*=10 films and television episodes)

Food/drink portrayed as triggers or causes of gout	Food/drink portrayed as protective or recommended for gout
Alcohol	Drink plenty of water
Apple pie	Fish
Bacon	Fruit and vegetables
Blood gravy	Omelette
Champagne	Salad
Chopped chicken liver	
Deli foods	
Drinkable cheese	
Garlic	
Lard cakes	
Liver balls	
Lung loaf	
Mackerel	
New York style food	
Organ meats: kidney, hearts, liver	
Pancakes	
Preserved fish like anchovies or herrings	
Red wine	
Rich food	
Skillet dripping	
Smothered chicken fried bacon	
Smothered chicken fried bananas	
Smothered pork chops	
Wine	

### Depictions of the management of gout

Management strategies for gout with examples are shown in Table 4. The majority of depictions in historical settings (17/19, 89%) and half of all depictions (22/44, 50%) showed no management strategies for gout. The most common management strategy, portrayed in 3/3 (100%) of depictions with historical settings and 5/19 (26%) with modern-day settings, was change in diet, for example in the Everybody Hates Chris episode ‘Everybody Hates the Gout’, in which the person with gout is handed raw vegetables and advised to “try not to eat any junk” (Everybody Hates Chris, S1Ep16, CBS).

**Table 4:** Management of gout in films and television episodes in which management was depicted (*n*=22 films and television episodes)

Management strategy	Total ***N***=22	Historical setting ***N***=3^**a**^	Modern-day setting ***N***=19	Example
Dietary management	8/22, 36%	3/3, 100%	5/19, 26%	**Doctor:** “What you need to do is quit pumping your boy full of purine-rich foods.” (King of the Hill, S3Ep18, Fox Network)
Analgesia	7/22, 32%	0, 0%	7/19, 37%	**Howard Wolowitz:** “I gave her enough pain meds to choke a … well, her.” (The Big Bang Theory, S7Ep9, CBS)
NSAIDs	3/22, 14%	0, 0%	3/19, 16%	**Dr Brenner**: “Well, gout usually responds better to aspirin” (Off the Map, S1Ep10, ABC)
Urate-lowering therapy	1/22, 5%	0, 0%	1/19, 5%	**Dr Ellingham:** “allopurinol … that’s a long-term solution.” (Doc Martin, S8Ep7, ITV)
Serum urate monitoring	1/22, 5%	0, 0%	1/19, 5%	**Dr Ellingham:** “test your blood to see if you’d benefit from allopurinol” (Doc Martin, S8Ep7, ITV)

^a^One episode had two characters with gout, these were counted separately

Analgesia was mentioned as a management strategy in 7/22 (32%) of films and television episodes depicting the treatment of gout, such as Doctors: “Can you inject him with something to numb it? Then we can cram his foot into his shoe” (Doctors, S11Ep234, BBC), and (non-steroidal) anti-inflammatory medications in 3/22 (14%), such as Dr. Ellingham in Doc Martin: “Yes, well, I’ll prescribe you naproxen to dull the pain” (Doc Martin, S8Ep7, ITV). Urate-lowering therapy and serum urate monitoring were mentioned in no depictions of gout in historical settings of gout and once in a modern-day setting.

### Humour and embarrassment

Gout was portrayed as humorous in 7/20 (35%) of depictions in historical settings and 19/24 (79%) of depictions in modern-day settings, such as in Gut Punch: Webster: “I thought only sailors get gout”, Scooter: “No, that’s scurvy. Gout’s a fat person disease” (Gut Punch, S1Ep1). In Bleak House, Lawrence Boythorn is amused by his neighbour’s gout: “Sir Arrogant Numskull is here, laid up with the gout! Ha, ha, ha, ha, ha, ha … serves him right.” (Bleak House, Ep4, BBC). It was shown as embarrassing in 11/20 (55%) of historical settings and 11/24 (46%) of modern-day settings (Table 5). An example is when Alf in Coronation Street has a flare of gout and complains to a customer: “Don’t broadcast it I said, it’s bad enough suffering it without having it shouted from the rooftops!” (Coronation Street, S1, Ep3641, ITV).

**Table 5:** Depictions of gout as humorous or embarrassing (*n*=44 films and television episodes)

Portrayal	Total (***N***=44)	Historical setting ***N***=20	Modern-day setting ***N***=24	Examples
Funny/humorous	26/44, 59%	7/20, 35%	19/24, 79%	**Howard Wolowitz:** “Oh, her gout’s flaring up. Turns out an apple pie a day does not keep the doctor away.” (The Big Bang Theory, S7Ep9, CBS)
				**Luci:** “Down the hatch, gouty. Keep this up and you’ll be gravely ill in no time.” (Disenchantment, S2Ep7, Netflix)
Embarrassing	22/44, 50%	11/20, 55%	11/24, 46%	**Richard Bucket:** “Gout! But that’s worse than a fungus infection.” (Keeping Up Appearances, S4Ep9, BBC)

## Discussion

In this study, we examined how gout is depicted in contemporary film and television. Gout is frequently used as a narrative device to denote nobility and to explain the absence of characters from important events. Gout is portrayed as painful, affecting mobility and preventing attendance at social activities, and is used to emphasise character flaws such as weakness, greed and overindulgence. The condition itself is shown as embarrassing and a source of humour. These findings reflect cultural beliefs about gout as a self-inflicted illness of dietary excess.

Some differences were seen in depictions of gout in historical and modern-day settings. Characters portrayed in modern-day settings were less likely to be members of the nobility, but more likely to be shown in the context of a comedy or humorous depiction of gout (79% versus 35%). These portrayals fit with the historical narratives about gout as a disease of the upper classes and associated with overindulgence [5]. Gout is frequently portrayed as comedic in modern-day content, reflecting the persistence of longstanding narratives about gout as caused by a lack of self-control.

Gout was commonly utilised as a plot device to explain the absence of characters from events, to set the scene of nobility and to explain the absence of characters from important social events or work. This is an accurate portrayal of the real-life experience of patients with gout, as absence from important events or work is frequently described by patients in qualitative studies [20, 21].

This study aligns with the results of a previous study from our group analysing descriptions of gout in contemporary newspaper articles, which similarly showed that gout was portrayed as a self-inflicted condition associated with lifestyle choices [22]. There is a possibility that by reinforcing a perception that gout is self-inflicted, and by omitting portrayals of effective treatments, these depictions may contribute to the undertreatment of gout [6]. Other studies have found inaccurate portrayals of other medical conditions, such as inflated rates of survival with CPR in popular television episodes, which similarly run the risk of perpetuating misconceptions and influencing the public’s medical decisions [23]. Although a direct link between the portrayal of gout in film and television and behaviour has not yet been studied, it has been shown that film or television portrayals can impact behaviour in other situations, including alcohol consumption, disordered eating, and cigarette smoking [24–26].

Limitations of the study include restricting the content to English language films and television episodes that aired from 1990. The study findings may not be generalisable to depictions of gout in non-English speaking countries. The time period was chosen to allow analysis of contemporary depictions that may influence current views about the illness. However, it is possible that content aired in earlier decades may influence the views of older people about the gout. The search term ‘gout’ may not have identified all depictions of gout; in order to maximise search results, film and television episodes were found through several methods including searching the Internet Movie Database, a Google search, and seeking recommendations from the Gout, Hyperuricemia and Crystal-Associated Disease Network (G-CAN). The films and television episodes were coded by two researchers to improve accuracy and discordant coding was solved by discussion with a third coder.

This study has shown that in contemporary film and television, gout is portrayed as a humorous and embarrassing condition, caused by dietary over-indulgence. Aligned with causal factors, dietary change and short term medication are portrayed as the major management strategies. These representations may influence societal beliefs about the illness, and reinforce inaccurate beliefs about the causes of gout and its management.

## Conclusion

The present study identified that in contemporary film and television, gout is portrayed as a humorous and embarrassing condition, caused by dietary indulgence. These depictions may reinforce inaccurate beliefs about the causes of gout and its management.

## Supplementary Information

**Additional file 1.** Television episodes included in analysis.

## Data Availability

The dataset used during the current study is available from the corresponding author on reasonable request.

## Abbreviations

G-CAN: Gout and Crystal-Associated Disease Network
MSU: Monosodium urate
IMDb: Internet Movie Database
