# Supplementary material for: “An apple pie a day does not keep the doctor away”: Fictional depictions of gout in contemporary film and television
Source: BMC Rheumatol. 2021 Jan 18;5:4. doi: 10.1186/s41927-020-00174-z (PMC7812654; doi:10.1186/s41927-020-00174-z)
Supplement: Supplementary file 1 — Additional file 1. Television episodes included in analysis. [file 41927_2020_174_MOESM1_ESM.docx]

**Supplementary material**

**Television episodes included in analysis**

1. Daniels G, Schur M, Hiscock N, Muharrar A. Parks and Recreation. Season 5, episode 20. Jerry’s Retirement [Television series episode]. New York City, NY: National Broadcasting Company (NBC); 2013.
2. Davies A, Dickens C, Chadwick J, White S. Bleak House. Episode 4 [Television series episode]. London: British Broadcasting Corporation (BBC); 2005.
3. Davies A, Dickens C, Chadwick J, White S. Bleak House. Episode 15 [Television series episode]. London: British Broadcasting Corporation (BBC); 2005.
4. Marlowe AW, Beall E, Francis S, Roe B. Castle. Season 4, episode 20. The Limey [Television series episode]. New York City, NY: American Broadcasting Company (ABC); 2012.
5. Warren T, Hill, B. Summers J. Coronation Street. Season 1, episode 3641 [Television series episode]. Salford, Greater Manchester: ITV Granada; 1994.
6. Groening M, Weinstein J, Horsted E, Angell J, Rowe J, Takeuchi S, Saleem J, Au DD, Sherak I. Disenchantment. Season 2, episode 7. Love’s Slimy Embrace [Television series episode]. Scotts Valley, CA: Netflix; 2019.
7. Stoneman R, Minghella D, Ferguson C, Crowdy M, Cole N. Doc Martin. Season 8, episode 7. Blade on the Feather [Television series episode]. London: ITV Studios; 2017.
8. Stoneman R, Minghella D, Ferguson C, Crowdy M, Cole N. Doc Martin. Season 3, episode 1. The Apple Doesn’t Fall [Television series episode]. London: ITV Studios; 2007.
9. Tindall J, Fallis B. Doctors. Season 11, episode 234. Mother’s help [Television series episode]. London: British Broadcasting Corporation (BBC); 2010.
10. Rock C, LeRoi A, Levine J. Everybody Hates Chris. Season 3, episode 2. Everybody Hates Caruso [Television series episode]. New York City, NY: Chris Rock Entertainment/ CBS Paramount Domestic Television; 2007.
11. Rock C, LeRoi A, Levine J. Everybody Hates Chris. Season 1, episode 16. Everybody Hates the Gout [Television series episode]. New York City, NY: Chris Rock Entertainment/ CBS Paramount Domestic Television; 2006.
12. Crane D, Kauffman M, Reich A, Cohen T, Weiss B. Friends. Season 9, episode 22. The One with the Donor [Television series episode]. New York City, NY: National Broadcasting Company (NBC); 2003.
13. Martin GRR, Benioff D, Weiss DB, Graves A. Game of Thrones. Season 4, episode 2. The Lion and the Rose [Television series episode]. New York City, NY: Home Box Office (HBO); 2014.
14. Martin GRR, Benioff D, Weiss DB, Graves A. Game of Thrones. Season 6, episode 1. The Red Woman [Television series episode]. New York City, NY: Home Box Office (HBO); 2016.
15. Chen W, Kitch K, Miller C, Tucci A, Miller C. Gout. Gut Punch [Online series episode]. Online: Gut Punch; 2009.
16. Eyre R., Shakespeare W. Henry IV, Part 2. The Hollow Crown [Television series episode]. London: British Broadcasting Corporation (BBC); 2013.
17. Shore D, Singer B. House. Season 1, episode 3. Occam’s Razor [Television series episode]. Los Angeles, CA: Fox Broadcasting Company; 2004.
18. Clarke R, Snoad H. Keeping Up Appearances. Season 4, episode 9. Angel Gabriel Blue [Television series episode]. London: British Broadcasting Corporation (BBC); 1994.
19. Judge M, Daniels G, Altschuler J, Krinsky D, Kuhlman A, Archer W. King of the Hill. Season 3, episode 18. Love Hurts and So Does Art [Television series episode]. New York City, NY: Fox Broadcasting Company; 1999.
20. Keeso J, Tierney J. Letterkenny. Season 5, episode 4. Spelling Bee [Television series episode]. Los Angeles, CA: New Metric Media/ Crave/ Hulu; 2018.
21. Fusco J, Chernuchin MS, Sakharov A. Marco Polo. Season 1, episode 3. Feast. [Television series episode]. Scotts Valley, CA: Netflix; 2014.
22. Bans J, Sachs J, Schelhaas L, Mohamed R, Zisk R. Off the Map. Season 1, episode 10. I’m Home [Television series episode] New York City, NY: American Broadcasting Company (ABC); 2011.
23. Logan J, Kirk B. Penny Dreadful. Season 2, episode 3. The Nightcomers [Television series episode]. London/ Los Angeles, CA: Neal Street Productions/ Desert Wolf Productions/ Showtime Networks; 2015.
24. Wooten J, Cherry M, Trainer D. The 5 Mrs. Buchanans. Season 1, episode 2. The Other Woman [Television series episode]. New York City, NY: Columbia Broadcasting System (CBS); 1994.
25. Lorre C, Prady B, Molaro S, Reynolds J, Howe J, Kaplan E, Holland S, Ferrari M, Del Broccolo A, Hernandez T, Cendrowski M. The Big Bang Theory. Season 7, episode 9. The Thanksgiving Decoupling [Television series episode]. New York City, NY: Columbia Broadcasting System (CBS); 2013.
26. Chase D. The Sopranos. Season 6, episode 21. Made in America [Television series episode]. New York City, NY: Home Box Office (HBO); 2007.
27. Hirst M, Podeswa J. The Tudors. Season 4, episode 8. As It Should Be [Television series episode]. Culver City, CA: Showtime/ Sony Pictures Television; 2010.
28. Carter C, Nielsen K, Follmer B, Van Allen B, Wong J. The X-Files. Season 11, episode 9. Nothing Lasts Forever [Television series episode]. Los Angeles, CA: Fox Broadcasting Company; 2018.
29. Ball A, Harris C, Buckner B, Lehmann M. True Blood. Season 1, episode 4. Escape from Dragon House [Television series episode]. New York City, NY: Home Box Office (HBO); 2008.

**Films included in analysis**

1. Wheatley B. A Field in England [Film]. London/ Austin, TX: Film4 Productions/ Drafthouse Cinema; 2013.
2. Braff Z. Going in Style [Film]. Burbank, CA: Warner Bros; 2017.
3. Hullum M. Lazer Team [Film]. Austin, TX: Rooster Teeth Productions; 2015.
4. Hudson H. My Life so Far [Film]. London: Enigma Productions; 1999.
5. Potter S. Orlando [Film]. San Francisco, CA: Adventure Pictures; 1992.
6. Campion J. The Portrait of a Lady [Film]. Los Angeles, CA: Polygram Filmed Entertainment; 1996.
7. Forster M. Stranger Than Fiction [Film]. Los Angeles, CA: Columbia Pictures; 2006.
8. Szabó I. Sunshine [Film]. Toronto: Alliance Atlantis Communications; 1999.
9. Dibb S. The Duchess [Film]. Los Angeles, CA: Paramount Vantage; 2008.
10. Lanthimos G. The Favourite [Film]. Los Angeles, CA: Fox Searchlight Pictures; 2018.
11. Landoval E. The One Misadventure of Dr. Gouty [Film]. Sand OVAL Productions; 2010.
12. Lord P. The Pirates! Band of Misfits [Film]. Los Angeles, CA: Columbia Pictures; 2012.
13. Apatow J. This is 40 [Film]. Los Angeles, CA: Apatow Productions; 2012.
14. Malle L. Vanya on 42^nd^ Street [Film]. London: Channel Four Films/ Mayfair Entertainment/ The Vanya Company; 1994.
15. Joffé R. Vatel [Film]. Paris: Légende Films; 2000.
